# Seroprevalence of the novel swine acute diarrhea syndrome coronavirus in China assessed by enzyme-linked immunosorbent assay

**DOI:** 10.3389/fcimb.2024.1367975

**Published:** 2024-04-26

**Authors:** Zuqing Liu, Ya Zhao, Jingyu Yang, Xi Liu, Yun Luo, Lili Zhu, Kun Huang, Feng Sheng, Xuezhu Du, Meilin Jin

**Affiliations:** ^1^ State Key Laboratory of Biocatalysis and Enzyme Engineering, School of Life Sciences, Hubei University, Wuhan, China; ^2^ National Key Laboratory of Agricultural Microbiology, Huazhong Agricultural University, Wuhan, China; ^3^ College of Veterinary Medicine, Huazhong Agricultural University, Wuhan, China; ^4^ Key Laboratory of Special Pathogens and Biosafety, Wuhan Institute of Virology, Chinese Academy of Sciences (CAS), Wuhan, China; ^5^ Hubei Jiangxia Laboratory, Wuhan, China

**Keywords:** coronavirus, SADS-CoV, S1 protein, ELISA, seroprevalence

## Abstract

The endemic outbreak of SADS-CoV has resulted in economic losses and potentially threatened the safety of China’s pig industry. The molecular epidemiology of SADS-CoV in pig herds has been investigated in many provinces in China. However, there are no data over a long-time span, and there is a lack of extensive serological surveys to assess the prevalence of SADS-CoV in Chinese swine herds since the discovery of SADS-CoV. In this study, an indirect anti-SADS-CoV IgG enzyme-linked immunosorbent assay (ELISA) based on the SADS-CoV S1 protein was established to investigate the seroprevalence of SADS-CoV in Chinese swine herds. Cross-reactivity assays, indirect immunofluorescence, and western blotting assays showed that the developed ELISA had excellent SADS-CoV specificity. In total, 12,978 pig serum samples from 29 provinces/municipalities/autonomous regions in China were tested from 2022 to 2023. The results showed that the general seroprevalence of SADS-CoV in China was 59.97%, with seroprevalence ranging from 16.7% to 77.12% in different provinces and from 42.61% to 68.45% in different months. SADS-CoV is widely prevalent in China, and its seroprevalence was higher in Northeast China, North China, and Central China than in other regions. Among the four seasons, the prevalence of SADS-CoV was the highest in spring and the lowest in autumn. The results of this study provide the general seroprevalence profile of SADS-CoV in China, facilitating the understanding of the prevalence of SADS-CoV in pigs. More importantly, this study is beneficial in formulating preventive and control measures for SADS-CoV and may provide directions for vaccine development.

## Introduction

Swine acute diarrhea syndrome coronavirus (SADS-CoV), also known as porcine enteric alphacoronavirus (PEAV) or swine enteric alphacoronavirus (SeACoV), was first detected and identified in China in 2017 (Gong et al., 2017; Pan et al., 2017; Zhou et al., 2018). SADS-CoV is the fourth porcine enteric coronavirus circulating in Chinese swine herds after transmissible gastroenteritis coronavirus (TGEV), porcine epidemic diarrhea virus (PEDV), and porcine delta coronavirus (PDCoV) (Wang et al., 2019; Liu and Wang, 2021; Turlewicz-Podbielska and Pomorska-Mól, 2021). The clinical symptoms caused by infection with SADS-CoV, including severe and acute diarrhea and acute vomiting, are similar to those caused by other known porcine enteric coronaviruses, with mortality rates of up to 90% in pigs under 5 days of age (Zhou et al., 2018; Xu et al., 2019). This novel porcine enteric coronavirus belongs to the genus *Alphacoronavirus* of the *Coronaviridae* family and is an enveloped, positive-sense, single-stranded RNA virus with a genome length of approximately 27 kb (Yang et al., 2020). SADS-CoV is closely related to bat coronavirus HKU2 and has 95% nucleotide homology with bat coronavirus HKU2 at the whole-genome level, indicating that the virus may have originated from bats (Zhou et al., 2018). SADS-CoV infects porcine cells and cells from several mammalian species, including primary human cells (Yang et al., 2019; Edwards et al., 2020; Luo et al., 2021; Mei et al., 2022). These studies highlight the risk of cross-species transmission of SADS-CoV and susceptibility to human infection (Yang et al., 2019; Edwards et al., 2020).

In January 2017, the first outbreak of SADS-CoV occurred on a pig farm in Guangdong Province and subsequently spread to three nearby pig farms, resulting in the deaths of nearly 25,000 piglets on four farms (Zhou et al., 2018). A retrospective survey showed that SADS-CoV first appeared in China in August 2016 (Zhou et al., 2019b). This was followed by outbreaks in southern China in 2019 and in Guangxi Province in 2021 (Zhou et al., 2019a; Sun et al., 2022). Molecular epidemiological surveys have shown that SADS-CoV infection is also present in other provinces, such as Beijing, Heilongjiang, Hebei, Shandong, Henan, Jiangxi, and Fujian, and the molecular prevalence of SADS-CoV ranges from 0.15% to 43.53% in clinical diarrhea samples from different places (Zhou et al., 2019b, 2020; Si et al., 2021; Zhang et al., 2022; Zhou et al., 2022). These findings suggest that SADS-CoV is widely spread in China. However, the seroprevalence of SADS-CoV in Chinese swine herds since the outbreak has not yet been described.

Coronaviruses enter the recipient cells through a specific interaction between the S protein on the surface of the virus particle and host receptor (Guan et al., 2020). The S protein determines the host range and tissue tropism of coronaviruses and is the main site of action for neutralizing antibodies during infection (Li et al., 2017; Yu et al., 2020). S proteins are homotrimers, with each subunit consisting of S1 and S2 structural domains (Yu et al., 2020). The S1 subunit generally contains the receptor binding domain (RBD), which is responsible for specific binding to the host receptor and is closely related to the formation of neutralizing antibodies (Guan et al., 2020; Yu et al., 2020). Compared with S proteins—in the context of diagnostic targets—S1 proteins have advantages, such as easy and high expression and superior sensitivity (Gimenez-Lirola et al., 2017; Yuan et al., 2019). Currently, the S1 protein has been used to develop diagnostic methods, including enzyme-linked immunosorbent assay (ELISA), for PEDV and PDCoV (Thachil et al., 2015; Lin et al., 2018). Studies have also been conducted to develop serodiagnostic methods utilizing the N proteins of porcine intestinal coronaviruses; however, it has been shown that the use of N proteins as serodiagnostic antigens exhibits some cross-reactivity among porcine intestinal coronaviruses (Gimenez-Lirola et al., 2017). Therefore, in this study, an ELISA based on the SADS-CoV S1 protein was established and utilized to test 12,978 porcine serum clinical samples from 29 provinces in China to study the seroprevalence of SADS-CoV. The results of this study provide the general seroprevalence profile of SADS-CoV for the first time and contribute to understanding the prevalence of SADS-CoV in China.

## Materials and methods

### Sample information

A total of 12,978 clinical porcine serum samples were collected between 2022 and 2023 across 29 provinces of China by the testing center of Wuhan Keqian Biological Co. Ltd. and stored for future use (**Table 1**). The SADS-CoV-positive serum was provided by the Wuhan Institute of Virology, Chinese Academy of Sciences. Furthermore, our laboratory stored 81 Specific Pathogen Free (SPF) swine sera. Additionally, our laboratory also preserved other porcine pathogen-positive serum samples including 4 PEDV, 5 TGEV, 5 African swine fever virus (ASFV), 5 porcine circovirus-2 (PCV-2), 6 porcine pseudorabies virus (PRV), 8 porcine reproductive and respiratory syndrome virus (PRRSV), 4 foot-and-mouth disease virus (FMDV), 3 porcine polioviruses (PPV), 3 Japanese encephalitis virus (JEV), and 6 classical swine fever viruses (CSFV).

**Table 1:** Seroprevalence of SADS-CoV from different provinces of China.

Province	No. of samples	No. of positive	Prevalence (%)
Heilongjiang	411	256	62.29
Jilin	188	139	73.94
Liaoning	281	192	68.33
Inner Mongolia	511	280	54.79
Shanxi	712	444	62.36
Hebei	421	260	61.76
Beijing	389	300	77.12
Tianjin	103	76	73.79
Xinjiang	247	148	59.92
Gansu	369	226	61.25
Ningxia	52	33	63.46
Shaanxi	415	214	51.57
Henan	760	450	59.21
Hubei	859	536	62.4
Hunan	890	570	64.05
Shandong	377	234	62.07
Jiangsu	301	155	51.5
Anhui	353	195	55.24
Zhejiang	673	407	60.48
Shanghai	48	8	16.7
Jiangxi	670	374	55.82
Fujian	460	253	55
Sichuan	761	448	58.87
Chongqing	478	325	67.99
Guizhou	476	252	52.94
Yunnan	407	214	52.58
Guangdong	697	410	58.82
Guangxi	596	345	57.89
Hainan	73	39	53.42
Total	12978	7783	59.97

### Cells and virus strain

Huh-7 cells were cultured in Dulbecco’s Minimal Essential Medium (DMEM; Invitrogen, Carlsbad, CA, USA) supplemented with 10% fetal bovine serum (FBS,LifeTechnologies) and 1% (w/v) antibiotics (penicillin,streptomycin;Invitrogen); Chinese hamster ovary (CHO) suspension cells were cultured using serum-free medium (Eminence). The SADS-CoV virus strain was isolated from piglet samples from the 2017 outbreak of piglet diarrhea in Guangdong, China, and preserved in the Microbial Virus Collection Center of Wuhan Institute of Virus Research, Chinese Academy of Sciences.

### Expression and purification of recombinant SADS-CoV N and S1 proteins

The SADS-CoV N gene was synthesized (Tsingke Biotechnology Co., Ltd.) and inserted into the PET-28a-His vector for prokaryotic expression. The modified vector was transformed into *E. coli* BL21 (DE3) cells, which were cultured overnight at 16°C in medium containing 1 mM IPTG. Bacteria were collected by centrifugation, resuspended in PBS, and fragmented by sonication. The N-terminal region, comprising 1–543 amino acids (SADS-CoV S1) of the SADS-CoV S protein containing the signal peptide, was synthesized and cloned into the pCMV-His expression vector to construct the recombinant plasmid pCMV-S1-His. The recombinant plasmid pCMV-S1-His was transfected into serum-free CHO cells using the polyetherimide (PEI) transfection reagent and cultured for 1 week. The supernatant was filtered through a 0.45 µm filter, collected, and purified with AKTA Pure 25 using Ni resin at 4°C. Proteins were subjected to gel filtration chromatography using a Superdex 200 Increase 10/300 GL column, and the protein concentration was determined using a G250 staining solution (Beyotime, Shanghai, China), following the manufacturer’s protocol.

### Western blotting

The purified recombinant SADS-CoV N and S1 proteins was analyzed via 7.5% sodium dodecyl sulfate-polyacrylamide gel electrophoresis (SDS-PAGE) and transferred onto a nitrocellulose membrane, which was blocked with 1% BSA in TBST for 1 h at 37°C. The nitrocellulose membranes were then incubated overnight with SADS-CoV-positive or negative serum as the primary antibody at 4°C. After incubation, the nitrocellulose membrane was washed thrice with TBST buffer for 10 min each and incubated with goat anti-swine enzyme-labeled secondary antibody (SouthernBiotech, USA) for 1 h at 37°C. Finally, the protein bands were detected using the ECL system (Advansta, San Jose, CA, USA).

### Indirect ELISA for the recombinant S1 protein

Specifically, 96-well ELISA plates were coated with 100 μL purified recombinant S1 protein in bicarbonate buffer (pH = 9.6) overnight at 4°C. The plates were washed thrice with PBST (PBS containing 0.05% Tween-20) and blocked in PBST containing 2% BSA for 2 h at 37°C. After washing the plates thrice, 100μL pig serum sample diluted with sample diluent (PBST containing 2% BSA) was added and incubated for 1 h at 37°C. After washing with PBST five times, 100 μL horseradish peroxidase (HRP)-conjugated goat anti-swine IgG (SouthernBiotech, USA) diluted with secondary antibody protectant was added to the plates and incubated at 37°C for 30 min. After washing with PBST five times, 100 µL tetramethylbenzidine hydrogen peroxide (TMB) substrate solution was added to each well, and the color was developed in the dark at 37°C for 10 min, followed by the addition of 50 µL of stop solution. Finally, the reading was immediately recorded at 630 nm optical density using a microplate reader (TECAN, Switzerland).

The optimal dilution of recombinant protein S1 with enzyme-labeled secondary antibody was determined using the checkerboard method, in which the concentration of S1 protein was gradually diluted in the following sequence: 4, 2, 1, 0.5, and 0.25 µg/mL. The enzyme-labeled secondary antibody was serially diluted two-fold from 1:5000 to 1:40,000. The ratio of positive serum OD_630_ value to negative serum OD_630_ value (P/N value) was maximized as the best reaction condition. Simultaneously, the conditions of ELISA for the detection of porcine serum antibodies, such as serum sample dilution and incubation time, were optimized.

### Indirect immunofluorescence

Huh-7 cells (2.5×10^5^) were inoculated in 12-well plates, cultured overnight, and then infected with SADS-CoV at an MOI of 0.1. After incubation at 37°C for 24 h, cells infected with SADS-CoV were fixed with 4% paraformaldehyde for 15 min and then permeabilized with 0.01% Triton X-100 on ice for 15 min. Then 1% BSA was added to the cells such that they were submerged and incubated for 30 min, followed by incubation with ELISA-positive serum or ELISA-negative serum (1:50) 2 h at 37°C, which was followed by incubation with FITC-conjugated goat anti-porcine secondary antibody (1:1000) (SouthernBiotech, USA) for 1 h at 37°C. Cells were incubated with 4,6-diamidino-2-phenylindole (DAPI;1:1000) for 10 min, and the stained nuclei were visualized under a fluorescence microscope (Leica, German).

### Statistical analysis

Data were analyzed using GraphPad Prism 8 and Excel. All experiments were repeated at least thrice.

## Results

### Expression and immunogenicity of the SADS-CoV S1 protein

**Figure 1:**
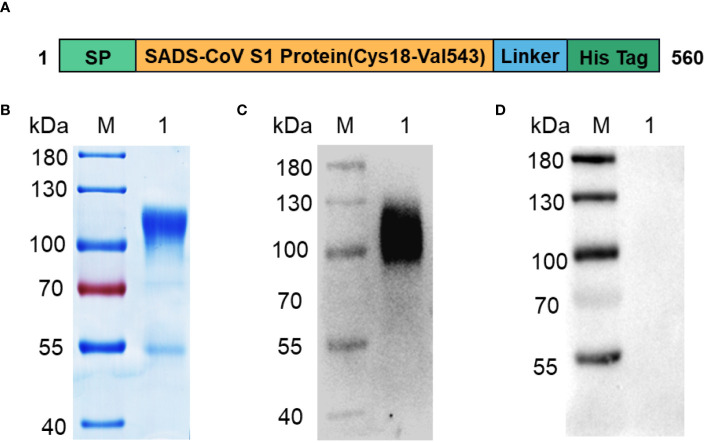
Expression and purification of recombinant SADS-CoV S1-His protein. **(A)** Design scheme for the SADS-CoV S1-His protein. **(B)** SDS-PAGE analysis of recombinant SADS-CoV S1-His protein. **(C, D)** Western blot analysis of SADS-CoV S1-His protein for immunogenicity identification. Recombinant SADS-CoV S1-His protein reacts with SADS-CoV-positive serum **(C)** but not with SADS-CoV-negative serum **(D)**. M: protein marker;1: SADS-CoV S1.

SADS-CoV mediates receptor binding of the virus to host cells through the RBD of the S1 subunit of the S protein, and studies on the receptor-binding region of SADS-CoV are lacking. Therefore, we selected the S1 subunit of the SADS-CoV S protein as the target for diagnostic test development. A schematic view of the SADS-CoV S1 protein is shown in **Figure 1A**. The optimized signal peptide sequence was constructed at the N-terminus to ensure high secretion efficiency of the protein, and a His-tag was attached at the C-terminus to facilitate affinity purification of the protein. Recombinant SADS-CoV S1 was successfully expressed in CHO cell culture supernatants, and SDS-PAGE showed that the S1-His band migrated to the 100–130 kDa range under reducing conditions (**Figure 1B**). Western blotting results showed that the recombinant S1-His protein expressed via CHO cells could react with SADS-CoV-positive serum but not with SADS-CoV-negative serum (**Figures 1C, D**), indicating that the recombinant S1-His protein has excellent immunogenicity.

### Establishment of the S1-indirect ELISA method

**Figure 2:**
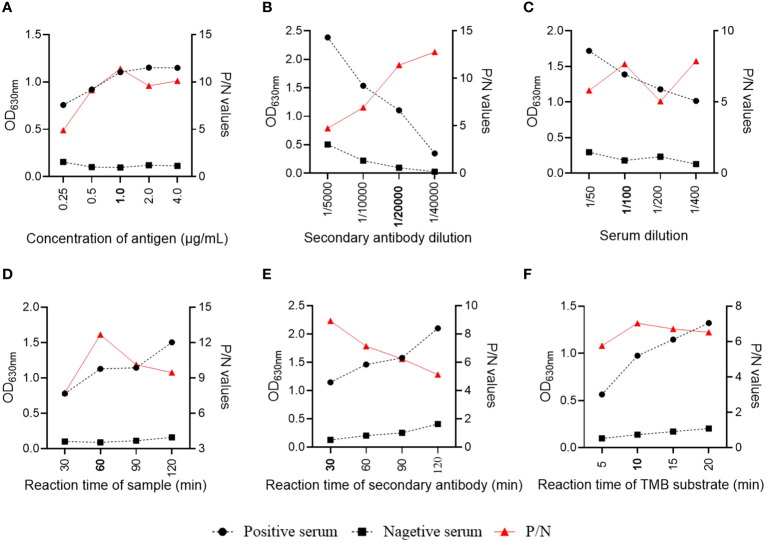
Optimization of S1-iELISA working conditions. **(A, B)** Checkerboard titration to determine the optimal concentration of encapsulated antigen and dilution of enzyme-labeled secondary antibody. **(C, D)** Serum sample dilution and reaction time. **(E, F)** Reaction time for enzyme-labeled secondary antibody and TMD substrate.

The assay conditions of the S1-iELISA were determined by checkerboard titration to maximize the SADS-CoV-positive to SADAS-CoV-negative serum ratio (P/N). The results showed that the dilution of the coating antigen S1 was 1 µg/mL, and the dilution of the enzyme-labeled secondary antibody was 1:20,000 when the P/N ratio was at the maximum (**Figures 2A, B**). Subsequently, we optimized the dilution of the serum samples and showed that the best discrimination between positive and negative serum samples was achieved when they were diluted 1:100 (**Figure 2C**). Finally, we optimized the reaction times of serum, enzyme-labeled secondary antibody, and tetramethylbenzidine hydrogen peroxide (TMB) substrate solution to achieve the best assay conditions. As shown in **Figures 2D–F**, the optimal reaction times for serum, enzyme-labeled secondary antibody, and TMB substrate solutions were 60 min, 30 min, and 10 min, respectively, when the ELISA conditions were optimized.

### Cut-off and reproducibility of the S1-iELISA

**Figure 3:**
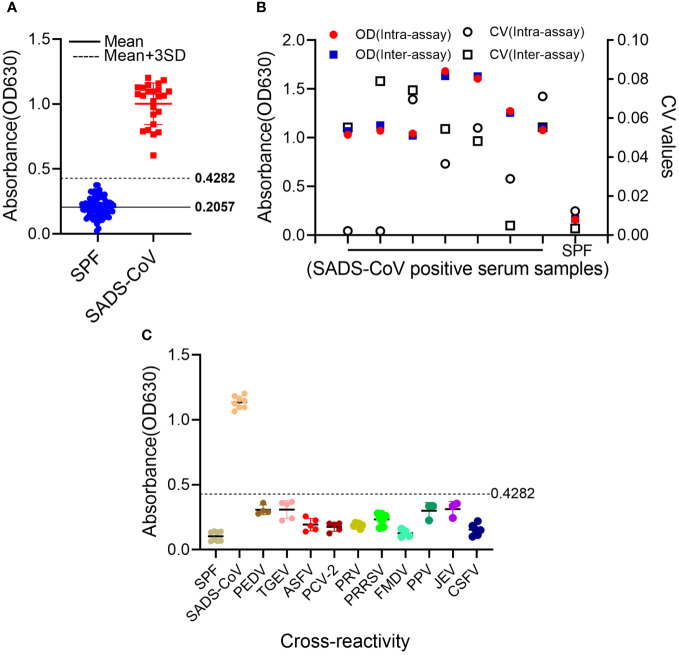
Determination of S1-iELISA cut-off values, specificity, and repeatability. **(A)** Determination of S1-iELISA cut-off values. S1-iELISA was used to test SADS-CoV-negative serum samples (n=81), and the cut-off value was calculated using the mean OD_630_ nm value of the SADS-CoV-negative serum samples plus three-fold SDs. Overall, 24 SADS-CoV-positive sera samples were used as controls. **(B)** S1-iELISA reproducibility analysis. Overall, eight sera samples were tested using the S1-iELISA, and CVs were calculated using the OD_630_ nm values of the serum samples to determine intra- and inter-assay reproducibility. **(C)** S1-iELISA specificity test. SADS-CoV-, PEDV-, TGEV-, ASFV-, PCV2-, PRV-, PRRSV-, FMDV-, PPV-, JEV-, and CSFV-positive sera were detected using the S1-iELISA, and the average OD_630_ nm value was calculated. The samples were judged to be positive or negative based on the cut-off value. SPF pig serum was used as a control.

In total, 81 porcine-negative serum samples were used to determine the cut-off value for the S1-iELISA. As shown in **Figure 3A**, the mean OD_630_ value of these negative samples was 0.2057, and the standard deviation (SD) was 0.0742. Therefore, the cut-off value for the S1-iELISA was 0.4282 (Mean+3SD), which indicates that SADS-CoV-positive sera were well distinguished from SADS-CoV-negative sera when the cut-off value was 0.4282.

The stability of S1-iELISA was assessed by determining intra- and inter-assay coefficient of variation (CV). The intra-assay CV of seven positive samples and one negative sample ranged from 0.21% to 7.12%; the inter-assay CV ranged from 0.35% to 7.9% (**Figure 3B**). Both intra-assay and inter-assay variabilities in the experiments were less than 10%, indicating the favorable reproducibility of S1-iELISA.

### Specificity validation of the S1-iELISA

To identify the specificity of the S1-iELISA, positive sera for common porcine pathogens were assayed, and the mean OD_630_ values of PEDV-, TGEV-, ASFV-, PCV-2-, PRV-, PRRSV-, FMDV-, PPV-, JEV-, and CSFV-positive samples were 0.3065, 0.3085, 0.1925, 0.1753, 0.1874, 0.2323, 0.1272, 0.2989, 0.3113, and 0.1539, respectively (**Figure 3C**). The OD_630_ values of these positive pathogen sera were all below 0.4282, indicating that these serum samples were SADS-CoV-seronegative and non-cross-reactive with this S1-iELISA. Therefore, the SADS-CoV S1-iELISA had excellent specificity.

**Figure 4:**
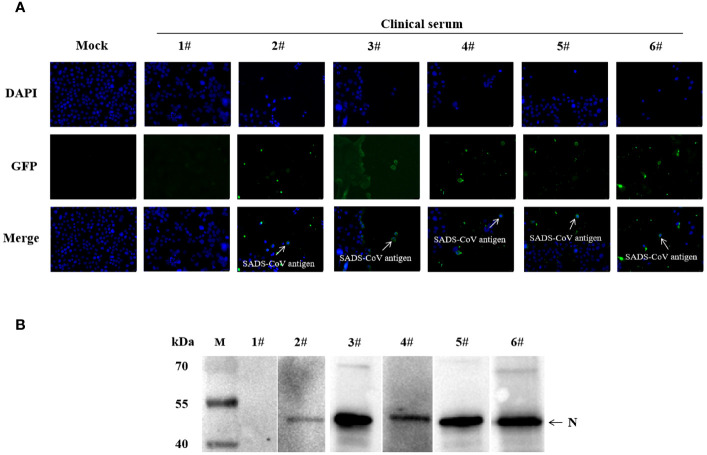
Validation of S1-iELISA sera by IFA and western blotting. **(A)** Validation of S1-iELISA serum by IFA. Huh-7 cells were inoculated in 12-well plates, cultured overnight, and then infected with SADS-CoV at MOI=0.1. After 24 h of infection, cells were co-incubated with ELISA-positive or ELISA-negative serum (1:50), followed by incubation with FITC-conjugated goat anti-swine antibody (1:1000). Cell nuclei were stained with DAPI (1:1000). Mock-inoculated Huh-7 cells were not infected with SADS-CoV. Scale bar: 20μm. **(B)** Validation of S1-iELISA serum by western blotting. 1#: Elisa-negative sera; 2#-6#: Elisa-positive sera.

To further validate the specificity of the S1-iELISA, we randomly selected five ELISA-positive sera samples and one ELISA-negative serum sample that had been tested by the S1-iELISA as primary antibodies for indirect immunofluorescence assay and western blotting. The IFA results showed that cells infected with SADS-CoV did not fluorescence when incubated with ELISA-negative serum, and cells infected with SADS-CoV showed green fluorescence when incubated with ELISA-positive serum (**Figure 4A**). In addition, western blotting results showed that the recombinant SADS-N protein displayed bands when incubated with ELISA-positive sera but not when incubated with ELISA-negative sera (**Figure 4B**). The above results indicate that S1-iELISA has excellent specificity.

### Seroprevalence of SADS-CoV in China

**Figure 5:**
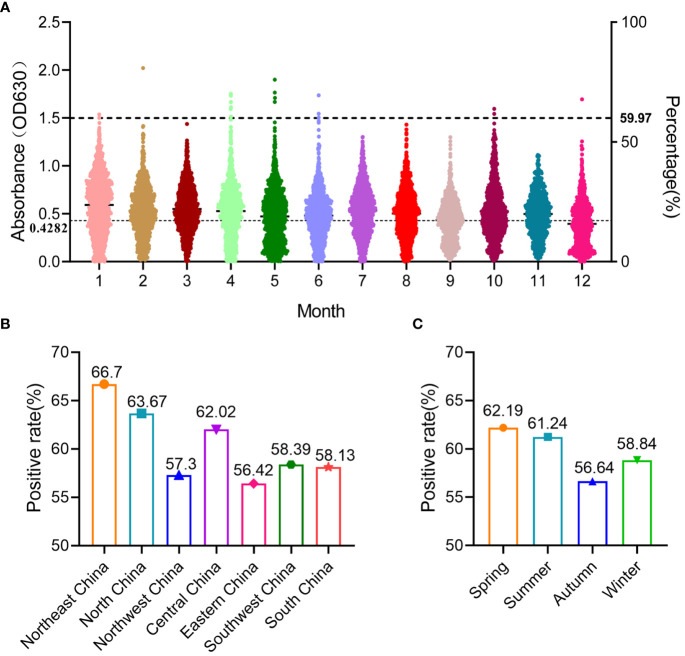
Prevalence of SADS-CoV in different months, regions, and seasons in China detected using S1-iELISA. **(A)** Prevalence of SADS-CoV in different month; 12,978 clinical porcine serum samples were collected from 29 provinces in China, from January 2022 to December 2022. **(B)** Prevalence of SADS-CoV across different regions in China. **(C)** Prevalence of SADS-CoV in different seasons.

In this study, 12,978 swine clinical serum samples were collected from 29 provinces/autonomous regions/municipalities in China between 2022 and 2023, and the general seroprevalence of SADS-CoV in China was 59.97% as determined using S1-iELISA (**Table 1**; **Figure 5A**). Among the 29 provinces/autonomous regions/municipalities where samples were collected, the seroprevalence ranged from 16.7% to 77.12% in different provinces (**Table 1**) and from 42.61% to 68.45% in different months (**Figure 5A**). The seroprevalence in Northeast China, North China, Northwest China, Central China, East China, Southwest China, and South China was 66.7% (587/880), 63.67% (1360/2136), 57.3% (621/1083), 62.02% (1556/2509), 56.42% (1626/2882), 58.39% (1239/2122), and 58.13% (794/1366), respectively (**Figure 5B**). The above results indicate that SADS-CoV is widely prevalent in Chinese swine herds, and that the seroprevalence of SADS-CoV is higher in Northeast, North, and Central China than in other regions. The seroprevalence rates of SADS-CoV in spring, summer, autumn, and winter were 62.19% (2158/3470), 61.24% (2313/3777), 56.64% (1552/2740), and 58.84% (1760/2991), respectively (**Figure 5C**). Compared to that in other seasons, SADS-CoV seroprevalence was the highest in spring, with a prevalence of 62.19%, and the lowest in fall, with a prevalence of 56.64%. Therefore, season may be a key factor influencing the prevalence and outbreak of SADS-CoV in Chinese swine herds.

## Discussion

Pig diarrhea is a widely prevalent problem in pig farming, which severely affects the development of the pig industry (Nan et al., 2021). Porcine enteric coronavirus is a common pathogen that causes porcine diarrhea and includes TGEV, PEDV, PDCoV, and SADS-CoV (Wang et al., 2019; Liu and Wang, 2021; Turlewicz-Podbielska and Pomorska-Mól, 2021). As a newly discovered porcine enteric coronavirus, SADS-CoV may be more harmful than other porcine enteric coronaviruses because it can cause several infections and deaths in a short time (Zhou et al., 2018). To date, no SADS-CoV vaccines or antiviral drugs have been developed. Therefore, the development of a rapid and reliable assay for SADS-CoV detection and epidemiological investigation of SADS-CoV infection are important for prevention.

SADS-CoV contains four main structural proteins, N, S, E, and M (Zhou et al., 2018). S-glycoprotein, the largest of the four structural proteins, is located on the surface of viral particles, has high immunogenicity, and plays an important role in SADS-CoV infection (Yang et al., 2020; Cao et al., 2023). The infection of susceptible cells by coronaviruses is mediated by the interaction of the S1 subunit of the S protein with host receptors and the fusion of the S2 subunit with the host cell membrane (Yu et al., 2020). Studies have shown that, in some porcine intestinal coronaviruses, S1 proteins are more immunogenic than the whole S protein and are the main inducers of antibodies (Yang et al., 2019). Therefore, the S1 subunit of the SADS-CoV S protein was selected as a diagnostic target in our study. Glycosylation of S1 proteins have an important impact on its immunogenicity (Guan et al., 2020; Yu et al., 2020; Peng et al., 2022). In contrast to the *E. coli* prokaryotic expression system, the mammalian cell eukaryotic expression system contains a range of post-translational modifications that produce proteins with glycosylation (Wurm, 2004). Therefore, the SADS-CoV S1 protein was expressed using a CHO cell expression system.

Clinical symptoms of SADS-CoV are similar to those of other known porcine enteric coronavirus infections and cannot be distinguished without laboratory clinical diagnosis (Peng et al., 2022). Thus, it is important to establish a specific serologic diagnostic method. In this study, the SADS-CoV S1 protein expressed by CHO cells was used as the antigen of the ELISA to detect positive sera associated with other swine pathogens, and the established ELISA showed no cross-reactivity, indicating that the S1-iELISA has exceptional specificity. In addition, the results of S1-iELISA serum validation by IFA and western blotting experiments further demonstrated that the S1-iELISA has preeminent specificity.

Epidemiologic investigation of SADS-CoV by S1-iELISA showed a prevalence of 59.97%, indicating that SADS-CoV infection is widespread in pigs across China. Although our study showed that SADS-CoV is widely prevalent in Chinese swine herds, there are few reports of large-scale deaths of pigs due to SADS-CoV infection, likely because: (1) SADS-CoV infection in pigs is a transient infection, and pigs can produce a limited amount of antibodies to clear SADS-CoV from their bodies; (2) most of the growing pigs infected with SADS-CoV do not show clinical symptoms or SADS-CoV has mutated during transmission to make it less pathogenic to piglets; and (3) in addition to this, high seroprevalence may be due to cross-reactivity with an unknown and less-pathogenic pathogen. However, these hypotheses need to be confirmed by further research. In another study, clinical porcine serum samples from 11 different provinces in China were tested in 2020 using S-iELISA, and up to 81.7% (246/300) of serum samples were positive (Peng et al., 2022). Epidemiologic findings of SADS-CoV using S-iELISA were considerably higher than our findings, which may be owing to the different sample sources, collection areas, and the different year of collection. Considering the widespread prevalence of SADS-CoV in Chinese swine herds, we should take some biological control measures to prevent its reemergence. In addition, it is necessary to strengthen continuous monitoring of this pathogen.

In conclusion, this study established an indirect ELISA with excellent specificity and stability based on the SADS-CoV S1 protein and utilized this method to conduct a large-scale seroepidemiological investigation of SADS-CoV in Chinese swine herds across a long-time span. The results showed that the general seroprevalence of SADS-CoV in China was 59.97%, and given the high seroprevalence of SADS-CoV, there is a strong need for SADS-CoV vaccine development to prevent piglet infection and mortality. Further research on SADS-CoV is needed, including the prevalence of co-infection of SADS-CoV with other porcine enteric coronaviruses and the development of an antiviral drug for SADS-CoV.

## Data availability statement

The original contributions presented in the study are included in the article/supplementary material. Further inquiries can be directed to the corresponding authors.

## Ethics statement

The animal studies were approved by The Institutional Animal Care and Use Committee at the Huazhong Agricultural University. The studies were conducted in accordance with the local legislation and institutional requirements. Written informed consent was obtained from the owners for the participation of their animals in this study.

## Author contributions

ZL: Writing – original draft, Investigation, Methodology, Project administration, Software. YZ: Writing – original draft, Data curation, Methodology, Project administration. JY: Writing – review & editing, Project administration, Software. XL: Writing – original draft, Project administration. YL: Project administration, Writing – review & editing. LZ: Project administration, Writing – review & editing. KH: Project administration, Writing – review & editing. FS: Project administration, Writing – review & editing. XD: Writing – review & editing, Conceptualization, Supervision. MJ: Writing – review & editing, Conceptualization, Funding acquisition, Supervision.
